# Brain dysconnectivity relates to disability and cognitive impairment in multiple sclerosis

**DOI:** 10.1002/hbm.25247

**Published:** 2020-11-26

**Authors:** Martin Sjøgård, Vincent Wens, Jeroen Van Schependom, Lars Costers, Marie D'hooghe, Miguel D'haeseleer, Mark Woolrich, Serge Goldman, Guy Nagels, Xavier De Tiège

**Affiliations:** ^1^ Laboratoire de Cartographie fonctionnelle du Cerveau UNI—ULB Neuroscience Institute, Université libre de Bruxelles (ULB) Brussels Belgium; ^2^ Department of Functional Neuroimaging, Service of Nuclear Medicine CUB—Hôpital Erasme, Université libre de Bruxelles (ULB) Brussels Belgium; ^3^ Center for Neurosciences Vrije Universiteit Brussel Brussels Belgium; ^4^ National MS Center Belgium; ^5^ Oxford Centre for Human Brain Activity, Wellcome Centre for Integrative Neuroimaging, Department of Psychiatry University of Oxford Oxford UK; ^6^ St Edmund Hall University of Oxford Oxford UK

**Keywords:** cognitive fatigue, cognitive impairment, functional connectivity, magnetoencephalography, multiple sclerosis

## Abstract

The pathophysiology of cognitive dysfunction in multiple sclerosis (MS) is still unclear. This magnetoencephalography (MEG) study investigates the impact of MS on brain resting‐state functional connectivity (rsFC) and its relationship to disability and cognitive impairment. We investigated rsFC based on power envelope correlation within and between different frequency bands, in a large cohort of participants consisting of 99 MS patients and 47 healthy subjects. Correlations were investigated between rsFC and outcomes on disability, disease duration and 7 neuropsychological scores within each group, while stringently correcting for multiple comparisons and possible confounding factors. Specific dysconnections correlating with MS‐induced physical disability and disease duration were found within the sensorimotor and language networks, respectively. Global network‐level reductions in within‐ and cross‐network rsFC were observed in the default‐mode network. Healthy subjects and patients significantly differed in their scores on cognitive fatigue and verbal fluency. Healthy subjects and patients showed different correlation patterns between rsFC and cognitive fatigue or verbal fluency, both of which involved a shift in patients from the posterior default‐mode network to the language network. Introducing electrophysiological rsFC in a regression model of verbal fluency and cognitive fatigue in MS patients significantly increased the explained variance compared to a regression limited to structural MRI markers (relative thalamic volume and lesion load). This MEG study demonstrates that MS induces distinct changes in the resting‐state functional brain architecture that relate to disability, disease duration and specific cognitive functioning alterations. It highlights the potential value of electrophysiological intrinsic rsFC for monitoring the cognitive impairment in patients with MS.

## Graphical abstract

This MEG study demonstrates that MS induces distinct changes in the resting‐state functional brain architecture that relate specifically to disability, disease duration and specific cognitive functioning alterations. It highlights the potential value of electrophysiological intrinsic rsFC for monitoring the cognitive impairment in patients with MS.

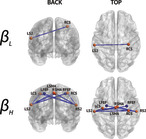

## 1 INTRODUCTION

Multiple sclerosis (MS) is a chronic, autoimmune, inflammatory, demyelinating but also degenerative disorder that affects both the white and gray matters of the CNS (for reviews, see, e.g., Compston & Coles, 2008; Geurts, Calabrese, Fisher, & Rudick, 2012; Ciccarelli et al., 2014). MS is the leading cause of nontraumatic neurological disability in young adults, especially in women. Neurological deficits are typically heterogeneous across patients, but usually affect motor, sensory, and autonomic functions (Compston & Coles, 2008).

More than 50% of patients with MS are also affected by cognitive impairments (CIs) and fatigue. CIs are mainly characterized by alterations in executive, attentional and memory functions, and are encountered throughout all disease stages (for a review, see, e.g., Chiaravalloti & DeLuca, 2008). Fatigue is defined as a lack of motivation, an overall feeling of exhaustion and behavioral performance decrements. It is divided into motor, psychosocial, and cognitive fatigue (for a review, see, e.g., Linnhoff, Fiene, Heinze, & Zaehle, 2019). Both CI and fatigue are important contributors to employment status, quality of life, and social functioning in patients with MS. Pharmacological and rehabilitation strategies are currently insufficient to alleviate these symptoms and require the development of novel therapeutic approaches (for a review, see, e.g., Benedict & Zivadinov, 2011). Providing a better understanding of the mechanisms involved in CIs and fatigue in MS is therefore of major importance (Di Filippo, Portaccio, Mancini, & Calabresi, 2018).

Although structural neuroimaging based on, for example, cerebral MRI, has been used extensively in the diagnosis and monitoring of MS (Polman et al., 2011), it has failed at explaining the degree and the variety of CIs observed in this disorder (Mollison et al., 2017). Apart from a link with gray matter atrophy, only weak associations have indeed been reproducibly found between structural MRI parameters and CIs/fatigue (Andreasen et al., 2019). Functional neuroimaging therefore offers a unique opportunity to better understand the pathophysiology of cognitive and fatigue symptoms in MS (for a review, see Van Schependom & Nagels, 2017).

MS has traditionally been considered as a disease affecting white matter tracts forming the structural connections between CNS gray matter structures (Compston & Coles, 2008). MS‐related gray matter involvement has also been clearly established (Mandolesi et al., 2015). To characterize the functional changes that accompany MS‐related alterations in *structural* white matter connectivity and gray matter lesions, imaging *functional* brain connectivity appears highly relevant to better understand the brain‐behavior relationship in this major brain disorder (Di Filippo et al., 2018). In a clinically heterogeneous disorder like MS, investigating functional brain connectivity at rest (i.e., in the absence of any goal directed task) has some key advantages over task‐based studies, that is, it is free of any performance bias and requires minimal patient cooperation, no task‐related training beforehand, and no complex experimental paradigm. Furthermore, previous studies have demonstrated a strong anatomical correspondence between task‐based and resting‐state functional connectivity (rsFC) (Cole, Bassett, Power, Braver, & Petersen, 2014; Mennes, Kelly, Colcombe, Xavier Castellanos, & Milham, 2013).

Functional MRI (fMRI) is the most widely used technique to investigate rsFC both in healthy subjects and patients with brain disorders. Previous fMRI studies have demonstrated that, at rest, the human brain is characterized by a high degree of spatial organization into segregated resting‐state networks (RSNs) (for a review, see, e.g., Deco & Corbetta, 2011). In MS, alterations in two RSNs have mainly been reported, that is, the default‐mode network (DMN) and the sensorimotor network (SMN). Several resting‐state fMRI (rs‐fMRI) studies have indeed shown changes (either increases or decreases, see Section 4 for further details) in DMN fMRI rsFC that were related to altered cognitive performance (Faivre et al., 2012; Hawellek, Hipp, Lewis, Corbetta, & Engel, 2011; Leavitt, Wylie, Girgis, DeLuca, & Chiaravalloti, 2014; Louapre et al., 2014; Rocca et al., 2010). Some studies have also shown altered fMRI rsFC within the SMN (Eijlers et al., 2017; Faivre et al., 2012; Janssen, Boster, Patterson, Abduljalil, & Prakash, 2013; Richiardi et al., 2012; Rocca et al., 2012; Roosendaal et al., 2010; Sbardella, Petsas, Tona, & Pantano, 2015), with some relationship between patients disability and SMN fMRI rsFC (Eijlers et al., 2017; Janssen et al., 2013).

**FIGURE 1:**
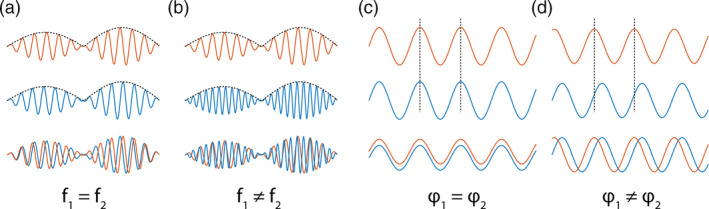
Illustration of envelope and phase coupling. Each column shows two signals both separately (orange and blue; top and middle rows) and superimposed (bottom row). Envelope correlation (a,b): The two oscillations have correlated envelopes (black dotted curves). This can occur both when their carrying frequencies (f_1_, f_2_) are equal (within‐frequency coupling, a) or different (cross‐frequency coupling, b), and independently of any phase coupling. Phase locking (c,d): The two equal‐frequency oscillations exhibit a phase relationship (illustrated by vertical dotted lines). This can occur when their phases (φ_1_, φ_2_) are equal (c) or maintain a constant difference (d)

**FIGURE 2:**
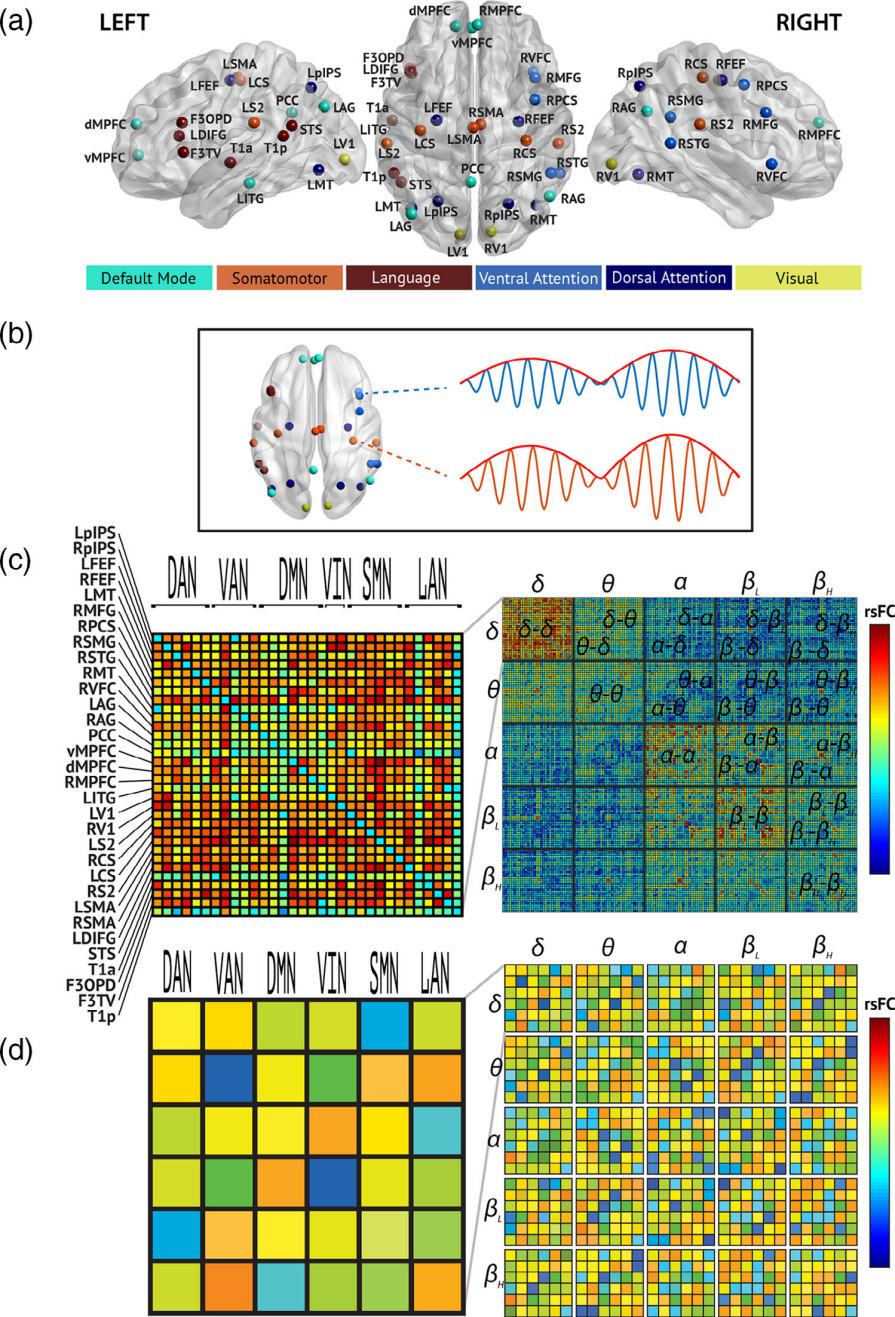
Illustration of the functional connectivity pipeline. (a). Overview of the locations and labels of the 32 nodes included in the connectome, color‐coded according to the network they belong to (see legend at the bottom). (b). Schematic illustration of the power envelope correlation used as rsFC measure. The envelope (red curve) of the neural oscillations at each node is used for the correlation analyses. (c). Matrix representation of all rsFC estimates across all node pairs (left) and all pairs of frequency bands, leading to the “multi‐layer” rsFC matrix as described in Brookes et al. (2016) (right). (d). As in Panel c, but here reporting the mean network rsFC within each network (diagonal entries) or across each network pair (offdiagonal entries). DAN, dorsal attention network; DMN, default‐mode network; LAN, language network; rsFC, resting‐state functional connectivity, RSN, resting‐state network; SMN, sensorimotor network; VAN, ventral attention network; VIN, visual network

While these results are valuable, MS‐related alterations in cerebrovascular reactivity might impact the neurovascular coupling at the basis of the fMRI signal (Marshall et al., 2014), potentially limiting the usefulness of fMRI in this disorder. Its low temporal resolution also precludes the study of neural oscillations, which support short‐ and long‐range functional brain connectivity and underlie a wide range of cognitive functions (for a review, see, e.g., Siegel, Donner, & Engel, 2012). Furthermore, fMRI failed to demonstrate alterations in neural network organization in patients with early MS, while electrophysiological investigations did in the same patients (Tewarie et al., 2015). For these reasons, investigations of electrophysiological, spectrally‐resolved rsFC with magnetoencephalography (MEG) or electroencephalography (EEG) have become increasingly popular (Hall, Robson, Morris, & Brookes, 2014). Previous EEG (Gschwind et al., 2016; Leocani et al., 2000; Van Schependom et al., 2014) and MEG (Cover et al., 2006; Schoonheim et al., 2013; Tewarie et al., 2013, 2014, 2015) studies have investigated rsFC alterations in patients with MS using phase‐based measures (i.e., synchronization between neural populations as assessed through the time‐delayed correlation of their oscillations or closely‐related measures, see Figure 1). Although a few studies suggested that phase‐based rsFC is related to fMRI‐based RSNs (Tewarie et al., 2016; Vidaurre et al., 2018), their main electrophysiological correlate is rsFC based on band‐limited power envelope correlation, that is, synchronization between neural populations as assessed through the correlation of the amplitude of their oscillations (Brookes et al., 2011; Colclough et al., 2017; de Pasquale et al., 2010; Garcés et al., 2016; Hipp, Hawellek, Corbetta, Siegel, & Engel, 2012; Hipp & Siegel, 2015; Liu, Farahibozorg, Porcaro, Wenderoth, & Mantini, 2017; Liu, Ganzetti, Wenderoth, & Mantini, 2018; Siems, Pape, Hipp, & Siegel, 2016; Tewarie et al., 2016; Wens et al., 2014; Zhang et al., 2009), see also Figures 1 and 2b. This rsFC index also has the critical advantage of being more robust on a test–retest basis than phase‐based measures (Colclough et al., 2016).

Although scarcely done with phase‐based rsFC (Tewarie et al., 2013), possible MS‐related reorganizations of electrophysiological RSNs estimated with envelope correlation, and their relationship with individual cognitive and clinical parameters, have not been assessed *per se*. Such investigation might prove crucial to achieve a better understanding of the brain‐behavior relationship in MS as it might involve specific alterations of within‐ and cross‐RSN interactions. This MEG study therefore investigates the impact of MS on human brain RSNs and its relationship with various factors (e.g., motor disability, disease duration, CIs, and fatigue) in a large population of patients with MS. For that purpose, we designed a comprehensive, prior‐free analysis of rsFC based on a functional parcellation of the human brain into major RSNs extended with investigations of both within‐ and cross‐frequency couplings as developed by Brookes et al. (2016). We hypothesized that MS would lead to definite alterations of rsFC within and between specific RSNs compared with matched healthy subjects, and that those changes would be associated with disability, CIs and fatigue.

## 2 METHODS

### 2.1 Participants

One hundred patients with MS (69 females, 31 males; age: 47.8 ± 9.8 years (mean ± SD) were recruited from the National MS Center Melsbroek with the following inclusion criteria: (i) diagnosis of MS according to the revised 2011 McDonald criteria (Polman et al., 2011), (ii) age between 18 and 60 years, (iii) disability score (Expanded Disability Status Scale; EDSS; Kurzke (1983)) ≤6.5, and (iv) no relapse or treatment with corticosteroids within the 6 weeks preceding participation to the study. Eighty‐five patients had relapsing–remitting MS, while 15 patients had progressive MS. We also recruited fifty‐four healthy subjects (33 females, 21 males; age: 47.5 ± 11.7 years) matched in terms of gender and age. For both groups, participants were excluded if they took recreational psychoactive drugs, had any implanted ferromagnetic materials and if they had any prior neurologic or psychiatric disorder (except MS in the patients' group). Twenty patients took benzodiazepines (such as alprazolam, clonazepam, flurazepam, lorazepam, or Triazolam) at the time of the study.

Data from 7 healthy controls and 1 patient were not included in the final analyses due to quality issues with the MRI (1 patient with MS, 1 healthy subject), MEG data (4 healthy subjects) and due to being severe outliers across the cognitive tests (2 healthy subjects). Therefore, ninety‐nine patients and forty‐seven healthy participants were included in the final analyses. Demographic and clinical details of the final included participants are presented in Table 1.

**TABLE 1:** Participants' demographic and clinical characteristics

	MS patients			Healthy subjects			*p*
Gender (f/m)	68/31			29/18			.501
MS subtype	RRMS	PPMS	SPMS				
	85	7	7				

	Mean/median	SD	Range	Mean	*SD*	Range	
Age (y, mean)	47.7	9.7	26–72	47.8	11.9	26–68	.94
Education (y, mean)	13.9	2.6	8–21	15.1	2.1	12/21	.003
Duration (y, mean)	16.1	9.4	1–54				
EDSS (median)	3		1–6.5				
Lesion load (ml, mean)	9.4	7.8	0.8–34.7				

*Note:* Mean, standard deviation (*SD*), and range (min–max) are given where appropriate (and just count for Gender), as well as a *p* value for the comparison between the two groups (age, education: unpaired Welch's *t* tests, gender: two‐sided *χ*^2^ test for two proportions). *Education*: total number of years in school since start of primary school. *EDSS*: Extended Disability Status Scale. *Duration*: disease duration, that is, number of years since initial diagnosis. *RR/PP/SPMS*: relapsing–remitting/primary progressive/secondary progressive multiple sclerosis.

The study was approved by the ethics committees of the Universitair Ziekenhuis Brussel (Commissie Medische Ethiek UZ Brussel, B.U.N. 143201423263, 2015/11) and the National MS Center Melsbroek (February 12, 2015). All participants gave their express written consent to participate in the study prior to their inclusion. Participants' consent was obtained according to the Declaration of Helsinki.

### 2.2 Neuropsychological and neurological evaluation

All participants underwent a neuropsychological evaluation performed by a trained test administrator immediately prior to the MEG acquisition. They were tested on information processing speed (The Signal Digit Modalities Test; SDMT; Smith, 1982), episodic memory (The California Verbal Learning Tests; CVLT; Delis, Kramer, Kaplan, & Ober, 2000), visuospatial memory (Brief Visuospatial Memory Test‐Revised; BVMT‐R; Benedict, 1997), verbal fluency (Controlled Oral Word Association Test; COWAT; Mitrushina, Boone, & D'Elia, 1998), motor (motor part of the Fatigue Scale for Motor and Cognitive Functions; FSMC; Penner et al., 2009), and cognitive fatigue (cognitive part of FSMC), as well as on upper extremity function (9‐Hole Peg Test; 9‐HPT; Mathiowetz et al., 1985). This led to a total of seven neuropsychological scores. Depressive symptoms were evaluated using the Beck Depression Inventory (BDI; Beck, Steer, & Brown, 1996). Additionally, experienced neurologists performed a standard EDSS test in patients with MS.

### 2.3 Data acquisition

Forty‐five participants (30 patients, 15 healthy subjects) were scanned on an Elekta Neuromag Vectorview™ neuromagnetometer (Elekta Oy, Helsinki, Finland), while the remaining 101 participants (69 patients, 32 healthy subjects) were scanned using a Triux™ system (MEGIN, Croton Healthcare, Helsinki, Finland) due to an upgrade of the MEG system. Both were placed in a light‐weight magnetically shielded room with a single mu‐metal/aluminum shell and active interference cancelation (Maxshield™, Elekta Oy, Helsinki, Finland; see De Tiège et al., 2008, for more details). Both systems featured the same whole‐scalp‐covering 306‐channel layout consisting of 102 sensor triplets, each composed of two orthogonal planar gradiometers and one magnetometer. There was no difference in acquisition parameters or MEG data processing between the two scanners. Of note, previous works that mixed Vectorview and Triux MEG recordings did not reveal any significant change in data quality, including for rsFC relying on power envelope correlation (Coquelet et al., 2020; Naeije et al., 2019). Recordings took place at the CUB Hôpital Erasme (Brussels, Belgium). Participants' anatomical fiducials (nasion and left and right preauricular areas) as well as about 400 head shape points were digitized using an electromagnetic tracker (Fastrak, Polhemus, Colchester, VT). They were equipped with four head tracking coils for continuous registration of head position while inside the MEG helmet. Neuromagnetic activity was recorded using active interference cancelation during 5 min of rest (eyes open, fixation cross, seated position, 0.1–330 Hz band‐pass filter, 1 kHz sampling rate).

All participants underwent a whole‐brain high‐resolution 3D T1‐weighted imaging (3T Achieva scanner, Philips Medical Systems, Best, The Netherlands) at a different hospital (Universitair Ziekenhuis Brussel, Brussels, Belgium) with the following acquisition parameters: repetition time (TR): 4.939 ms, echo time (TE): 316 ms, flip angle: 8°, field of view (FOV): 230 × 230 mm^2^, 310 sagittal slices resolution: 0.53 × 0.53 × 0.5 mm^3^. To estimate structural markers of neurodegeneration typically used in MS, a T2‐weighted imaging FLAIR sequence was also acquired (TR: 4800 ms, TE: 316 ms, inversion time: 1650 ms, FOV: 288 × 288 mm^2^, 321 slices, 1.12 mm slice thickness, resolution: 0.6 × 0.6 × 1.12 mm^3^). The median delay between the MRI and MEG sessions was 5 days.

### 2.4 MEG data preprocessing

The signal space separation algorithm (Taulu, Simola, & Kajola, 2005), as implemented in the proprietary software Maxfilter™ (MEGIN, Croton Healthcare, Helsinki, Finland, version 2.1 with default parameters), was used to subtract external magnetic interferences and correct for head movement. Note that total head displacement (i.e., total positional change of the head between start and end of acquisition) was not significantly different between patients and healthy participants (*t*_144_ = 0.5, *p* = .58). Signal components representing physiological noise artifacts (i.e., eye movements and blinks, cardiac artifacts) were removed by an independent component analysis (Vigário, Särelä, Jousmäki, Hämäläinen, & Oja, 2000) of downsampled (250 Hz) and band‐pass filtered (0.1–45 Hz) MEG data, and identified by inspection of their spatial topography, time course and frequency spectrum. The number of removed components was not significantly different between MS patients and healthy participants (mean: 4, range: 2–6 and 3–7, respectively, *t*_144_ = 0.32, *p* = .75). Downsampling, filtering, and ICA were all done using the OHBA Software Library (OSL, https://github.com/OHBA-analysis). The cleaned MEG data were finally filtered into five frequency bands: delta (**δ**, 1–4 Hz), theta (**θ**, 4–8 Hz), alpha (**α**, 8–12 Hz), low beta (**β**_**L**_, 12–21 Hz), and high beta (**β**_**H**_, 21–30 Hz).

### 2.5 Source reconstruction

The MEG forward model was computed for each participant based on their MRI, which was anatomically segmented using FreeSurfer (Fischl, 2012). MEG and MRI coordinate systems were manually coregistered within the proprietary software MRIlab™ (MEGIN, Croton Healthcare, Helsinki, Finland) based on the acquired anatomical fiducials and head‐surface points. A common source space (5‐mm rectangular grid) was defined in the Montreal Neurological Institute (MNI) brain volume and deformed onto the participants' MRIs using a nonlinear spatial normalization scheme (Ashburner & Friston, 1999) as implemented in SPM8 (Friston, 2006). Subject‐specific forward models were then computed using the single‐layer boundary element method implemented in MNE‐C (Gramfort et al., 2014).

Source projection of band‐specific MEG signals over a grid of the whole brain volume relied on minimum norm estimation (MNE, Dale & Sereno, 1993) based on the implementation detailed in Wens et al. (2015). The noise covariance was estimated from 5 min of empty room recordings and the regularization parameter was adapted to the MEG signal‐to‐noise ratio via the prior consistency condition derived in Wens et al. (2015). The reconstructed source time series were further projected onto their direction of maximum variance (Brookes et al., 2011; Wens et al., 2014).

### 2.6 Connectivity analysis

Our pipeline for intrinsic rsFC analysis is illustrated in Figure 2. For each participant, we constructed an all‐to‐all rsFC connectome comprising 32 nodes (Figure 2a) distributed across six canonical resting‐state networks defined *a priori* based on a meta‐analysis of fMRI rsFC and used in de Pasquale et al. (2012): the dorsal attention (DAN), the ventral attention (VAN), the default‐mode (DMN), the visual (VIN), the sensorimotor (SMN) and the language (LAN) networks. The 32‐node connectome was adapted from one used in de Pasquale et al. (2012), which comprised 42 nodes (the VIN, originally composed of 10 closely packed nodes in striate and extrastriate visual cortices, was reduced to two nodes in right and left primary visual cortices, while one subcortical node in each of the left and right putamen were removed as we only used cortical sources for our analysis). We then computed the slow envelope (i.e., Hilbert envelope low‐pass filtered to 1 Hz) correlation between the band‐specific source time courses of each node pair (which will be referred to as *nodewise* rsFC in the results; Figure 2b). Spatial leakage was reduced prior to rsFC computation using pairwise static orthogonalization (Brookes, Woolrich, & Barnes, 2012). We did not need to use multivariate symmetrical orthogonalization (Colclough, Brookes, Smith, & Woolrich, 2015) here because spatial leakage is inherently symmetrical with MNE source reconstruction (Hauk & Stenroos, 2014). To investigate both within‐ and cross‐frequency coupling, we used a “*multi‐layer*” network design (Brookes et al., 2016) by allowing the band of each node signal to be the same or different from each other (Figure 2c). To control for possible power‐induced effects in our rsFC, we also estimated source signal absolute power (i.e., their temporal variance) at each node with noise standardization to correct for the depth bias (Pascual‐Marqui, 2002). We focused here on absolute power to ensure that rsFC changes are not merely due to modulations in signal‐to‐noise ratio (Muthukumaraswamy & Singh, 2011), but it is noteworthy that relative power changes associated to peak frequency shifts have been reported in patients with MS (Schoonheim et al., 2019; Van der Meer et al., 2013).

Taking advantage of the nodes' classification into networks, we also estimated mean within‐ and cross‐network coupling by averaging rsFC values over appropriate node pairs (see, for example, de Pasquale et al., 2012), again both within and across frequency bands (*mean network* rsFC; Figure 2d). In this context, power estimates were also averaged within each network.

### 2.7 Statistical analyses

Neuropsychological and clinical test scores were compared between patients with MS and healthy subjects using two‐tailed unpaired *t* tests. Because of our unbalanced design (99 patients vs. 47 healthy subjects), we used the Welch's version of the *t* statistic throughout this work, as it is more resilient to population heterogeneity (Ruxton, 2006). The possible confounding effects of age, sex and educational level were regressed out beforehand within each group, as well as benzodiazepine status for patients. The significance level was set to *p* = .05 Bonferroni corrected for the number of tests, that is, 8. As the MS group consisted of patients with relapsing–remitting MS and patients with progressive MS, we also performed a control test to check whether there were significant differences between those two subgroups of patients with MS.

The difference in nodewise rsFC or mean network rsFC between patients and healthy subjects was assessed by a mass‐univariate statistical contrast of the two corresponding “multi‐layer” matrices. Specifically, we considered for each matrix entry (i.e., two nodes or networks in their respective frequency band) Welch's *t* statistic comparing the 99 rsFC values in patients and the 47 values in healthy subjects, from which the effect of several confounding factors was regressed out beforehand (7 regressors for patients and 6 for healthy subjects): power estimates of the two corresponding nodes or within‐network averages, age, sex, educational level, and MEG system type (Vectorview vs. Triux) for both groups of participants, and additionally benzodiazepine status for patients to mitigate the effect of this psychotropic drug on brain activity (see, for example, Van Schependom et al., 2019). Regressing out power avoids power‐induced rsFC changes (Muthukumaraswamy & Singh, 2011), and regressing out system type eliminates any possible effect related to the MEG system upgrade (notwithstanding the absence of important data quality changes, see Naeije et al., 2019; Coquelet et al., 2020). To perform maximum statistic testing (see below), the unpaired permutation distribution of *t* matrices was generated by randomly shuffling patients' and healthy subjects' nodewise/mean network rsFC matrices (after regression of the respective confounding factors).

Within each group, we also analyzed the correlation between nodewise/mean network rsFC and clinical or neuropsychological scores (patients: disease duration, as measured from first clinical diagnosis, and 9 behavioral scores (listed in Section 2.2); healthy subjects: 8 behavioral scores (the same as for patients except EDSS)). We estimated a multiple regression model of nodewise/mean network rsFC values with the score of interest and the confounding factors as regressors and extracted the regression coefficient ***β*** corresponding to the relevant score. The permutation distribution of the resulting ***β*** matrices was generated by randomly shuffling the participants' order (within each group) against their respective scores before the regression analysis.

Significance levels were established using two‐tailed maximum statistic testing (Nichols & Hayasaka, 2003) to simultaneously correct for the multiple comparisons across all pairs of nodes/networks and frequency band interactions. The null distribution of the maximum absolute value over all matrix entries was estimated from the permutation schemes described above (number of permutations: 10^5^), and a significance threshold at significance level *p* was defined as the (1–*p*)^th^ percentile of this permutation distribution. We set *p* = .05 Bonferroni corrected for the total number of tests, that is, 38 (1 contrast, 10 correlations for patients, and 8 correlations for healthy subjects = 19 tests, performed for both nodewise and mean network rsFC). All supra‐threshold values were deemed to exhibit a significant effect (Nichols & Hayasaka, 2003). The *p* value of each maximum statistic, that is, the null probability of exceeding the observed maximum absolute value, was also estimated with its permutation distribution.

We also performed posthoc analyses to further check our results. To confirm the absence of power differences that could drive rsFC contrasts, we applied an analogous statistical *t* contrast on power estimates (now within frequency only and without power regression). Additionally, to see whether correlations within the patients' and healthy subjects' groups themselves were significantly different, we examined the group contrast of the corresponding ***β*** coefficients. Their permutation distribution was obtained by shuffling participants (i.e., patients vs. healthy subjects) before regression, as for the *t* contrasts. These *β* contrasts were only investigated for clinical or neuropsychological scores exhibiting significant correlations. These extra analyses were performed at the same significance level as above (i.e., *p* = .05/38).

### 2.8 Structural versus functional modeling of CI

An important question brought by the analysis of MS‐related rsFC changes and their correlation to cognitive scores, is whether electrophysiological rsFC provides an added value to describe MS‐related CI compared to structural markers that are often used (e.g., brain atrophy, cortical lesions, see e.g., Van Schependom & Nagels, 2017). To address this issue, we focused on cognitive scores that were significantly altered in patients with MS, and we first built for each of them a purely structural regression model with two well‐established markers of neurostructural damage as dependent variables, that is, global lesion load and normalized thalamic volume (see e.g., Barkhof, 2002; Tewarie et al., 2013). They were obtained individually from 3D T1‐weighted imaging and FLAIR MRIs using lesion detection and tissue segmentation implemented in the icobrain software (version 3.1; for details, see Jain et al., 2015). We then considered similar regression models that further included functional electrophysiolgical markers, and assessed whether this led to a significant increase in the amount *R* ^2^ of explained variance. Specifically, we added as dependent variables all mean network connectivity values (across RSNs and frequency bands, see Figure 2d) exhibiting a significant MS‐related contrast. Given that structural and functional parameters are related (Tewarie et al., 2013; Van Schependom & Nagels, 2017), these rsFC variables were orthogonalized with respect to the structural parameters before being used in the regression. This merely amounts to a reparametrization that emphasizes the independent information brought by rsFC but leaves the *R* ^2^ statistic unchanged.

By design, the structuro‐functional regression model is biased toward a larger *R* ^2^ than the purely structural model as it contains more dependent variables. To assess whether rsFC actually increases the *R* ^2^ beyond this bias, we performed nonparametric statistical model comparisons. The permutation distribution of *R* ^2^ was generated under the null hypothesis that rsFC does not bring more information about patients' cognition than what is already entailed by structural markers, that is, that its orthogonal part is statistically independent of cognitive scores. Specifically, 10^4^ null samples of *R* ^2^ were obtained by randomly shuffling the patients' order in the orthogonalized rsFC parameters before estimating the regression model and its *R* ^2^ value. Importantly, this permutation approach avoids the aforementioned bias and preserves orthogonality with the structural parameters. The *p* value on the *R* ^2^ statistic was derived as the fraction of null samples exceeding the *R* ^2^ value of the original structuro‐functional regression model.

## 3 RESULTS

**FIGURE 3:**
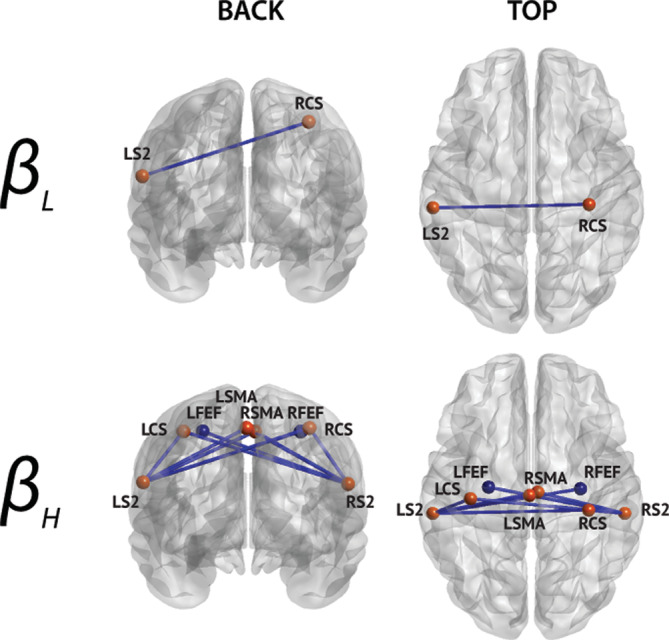
Significant nodewise rsFC changes between multiple sclerosis patients and healthy subjects. All are lower in patients. The bands in which significant differences were detected are indicated on the left. Only nodes with significant connections are shown (node color corresponds to their network, see Figure 2a), and their labels are superimposed

**FIGURE 4:**
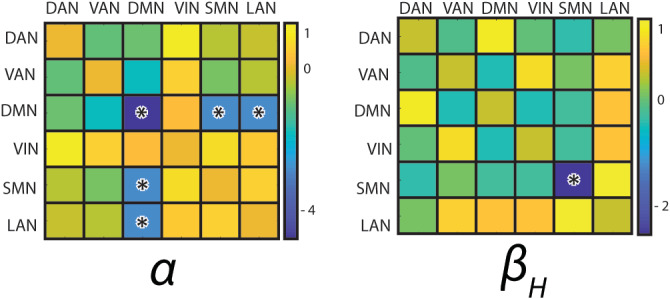
Significant mean network rsFC changes. Negative values indicate lower mean network rsFC in multiple sclerosis patients than in healthy subjects. Significant differences are indicated by asterisks (*p* < .05 corrected). We only show frequency bands exhibiting significant differences. DAN, dorsal attention network; DMN, default‐mode network; LAN, language network; SMN, sensorimotor network; VAN, ventral attention network; VIN, visual network

**FIGURE 5:**
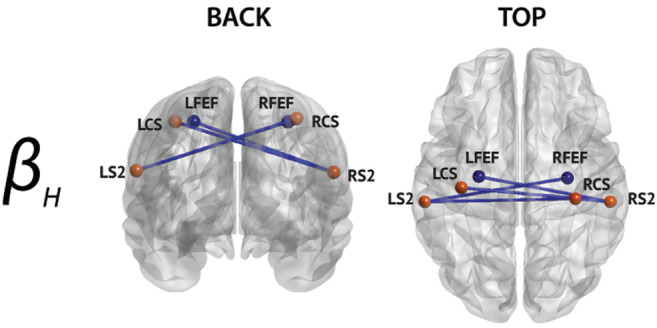
Connections significantly correlated with EDSS in multiple sclerosis patients (all correlations are negative). The band in which significant correlations were detected is indicated on the left. Only nodes with significant connections are shown (node color corresponds to their network, see Figure 2a), and their labels are superimposed

**FIGURE 6:**
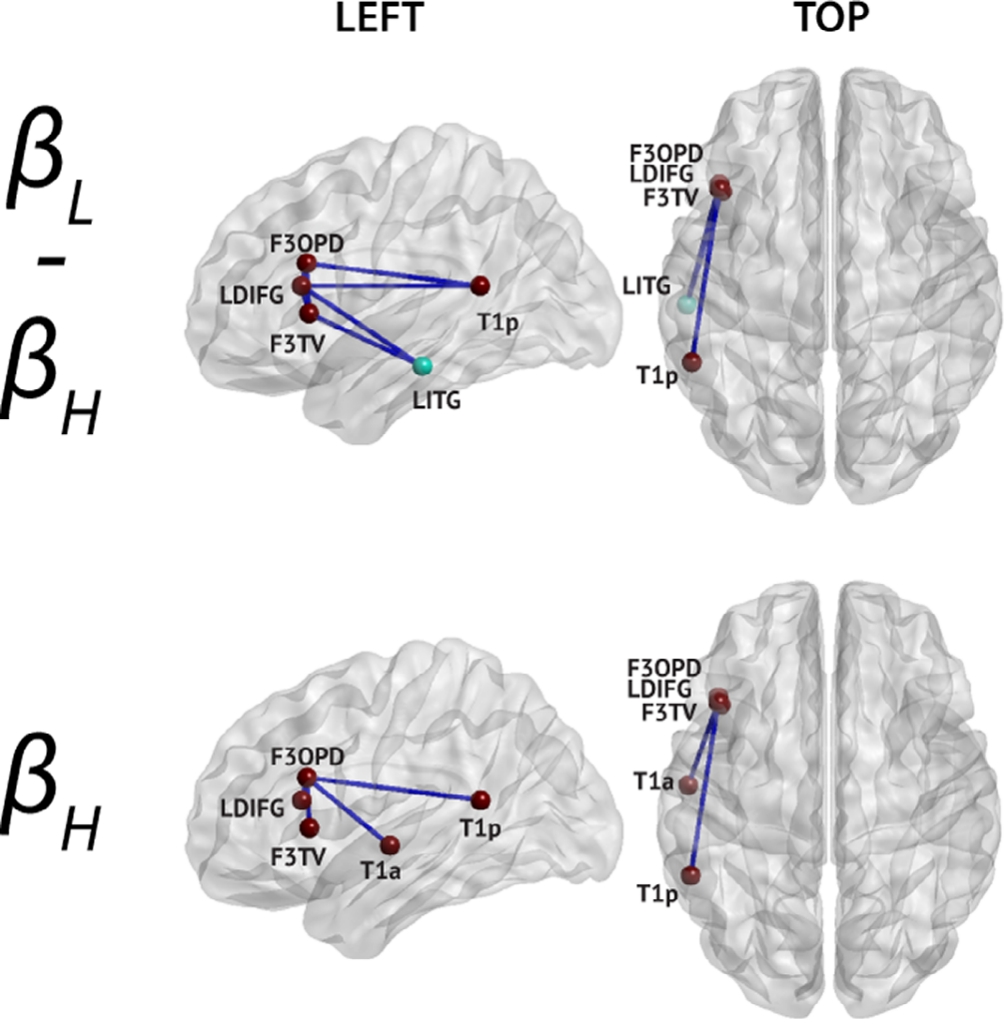
Connections disclosing significant correlation between rsFC and disease duration in patients with multiple sclerosis (all correlations are negative). The corresponding frequency bands are indicated on the left. Only nodes with significant connections are shown (node color corresponds to their network, see Figure 2a), and their labels are superimposed

**FIGURE 7:**
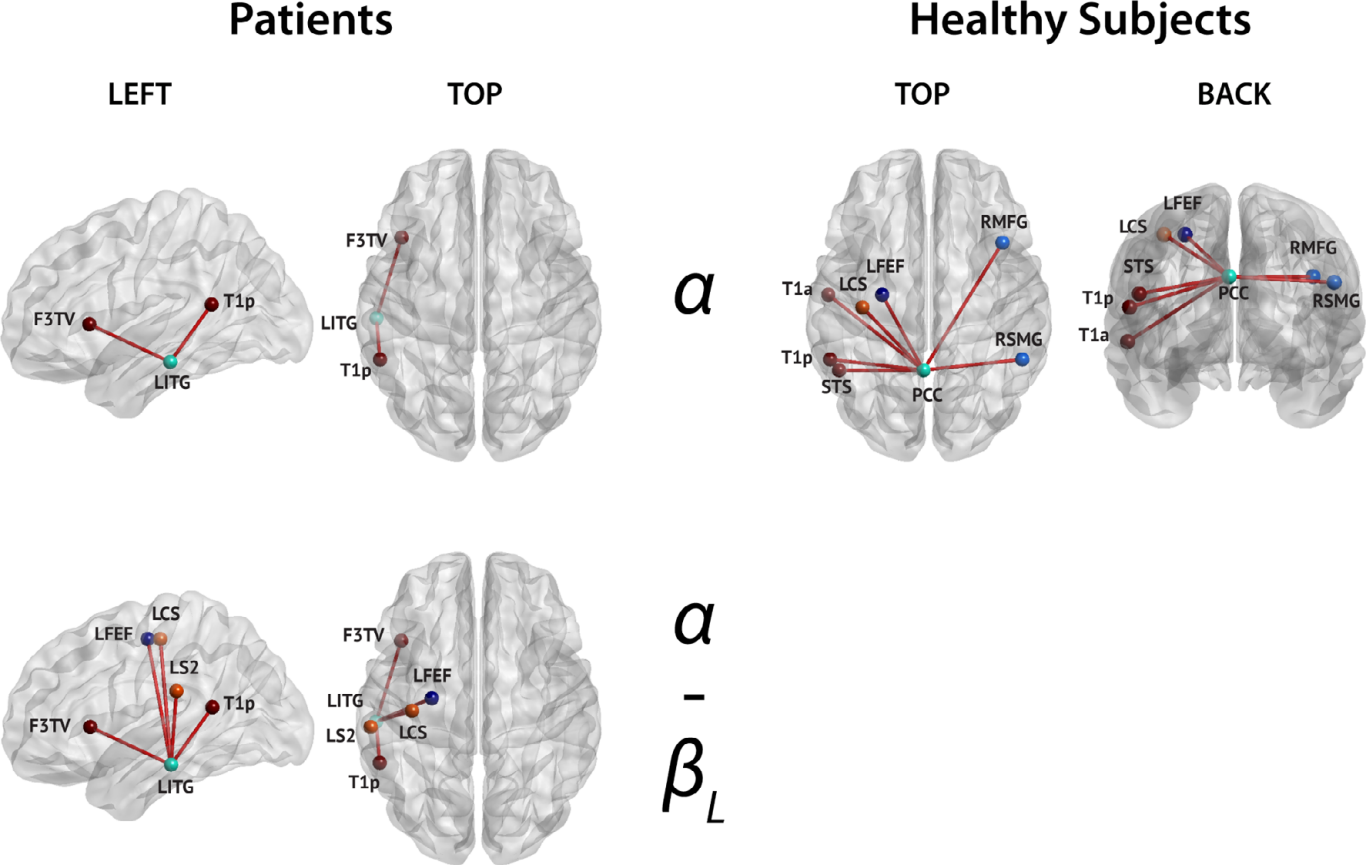
Connections disclosing significant correlation (all positive) between rsFC and verbal fluency for both multiple sclerosis patients (left) and healthy subjects (right). The corresponding bands are indicated in the middle. Only nodes with significant connections are shown (node color corresponds to their network, see Figure 2a), and their labels are superimposed

**FIGURE 8:**
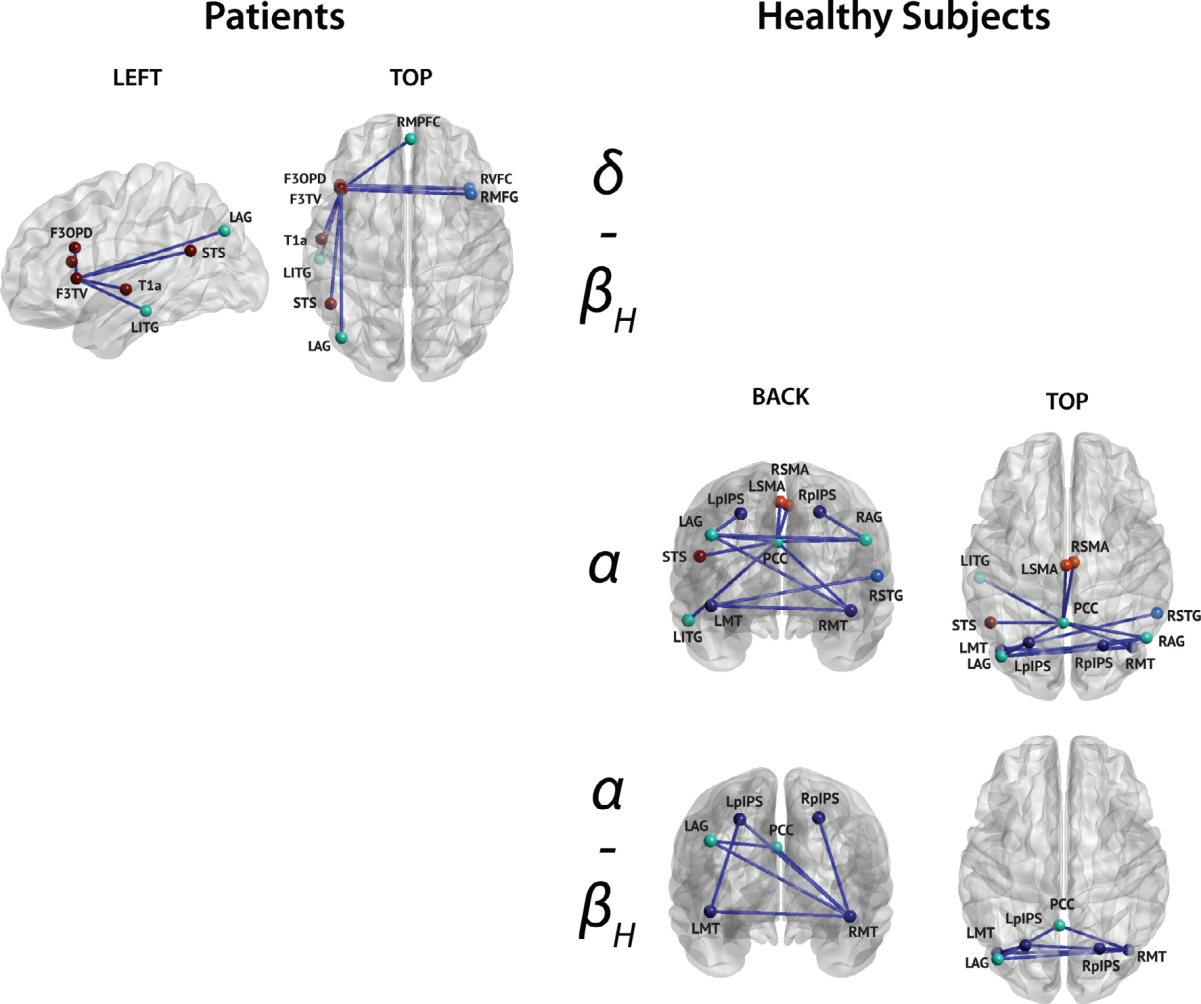
Connections disclosing significant correlation (all negatives) between rsFC and the cognitive fatigue score for both multiple sclerosis patients (left) and healthy subjects (right). The corresponding bands are indicated in the middle. Only nodes with significant connections are shown (node color corresponds to their network, see Figure 2a), and their labels are superimposed

We investigated the differences in electrophysiological power envelope rsFC among six major RSNs within and across five frequency bands (i.e., **δ**, **θ**, **α**, **β**_**L**_, and **β**_**H**_) in patients with MS and matched healthy subjects, both at node and mean network levels. Correlations between clinical/neuropsychological test scores and both nodewise and mean network rsFC were also performed in the two cohorts of participants. For all the results henceforth described (Figures 3, 4, 5, 6, 7, 8), significance was determined through a maximum‐statistic permutation approach after controlling for signal power, age, gender, education level, and MEG system type as well as benzodiazepine status for patients, with additional correction for the total number of tests that were performed. Importantly, only the significant results will be henceforth reported, that is, all other tests were nonsignificant. Among these, notably, no significant power differences were found in the contrast between patients with MS and healthy subjects (|*t*| < 1.2, permutation *p* value > .3) in any of the frequency bands.

### 3.1 Comparison of clinical and neuropsychological scores

The neuropsychological test scores revealed that verbal fluency was significantly lower (COWAT, *t* = −2.9, *p =* .0044), while both cognitive and physical fatigues were significantly higher (cognitive and motor part of FSMC, *t* = 6.9, *p* = 9.2 × 10^−11^ and *t* = 5.4, *p* = 6.5 × 10^−9^, respectively) in patients with MS compared to healthy subjects. Of note, there was no correlation between verbal fluency and cognitive or physical fatigue in neither patients nor healthy subjects (Pearson correlation test; *r* = .16, *p* = .13 and *r* = .12, *p* = .32, respectively), while cognitive and physical fatigue were similarly and strongly correlated in both groups (*r* = .78, *p* = 2.06 × 10^−10^ and *r* = .76, *p* = 1.7 × 10^−8^). None of the other scores were significantly different between groups after correction for the number of tests, including depression as assessed by the BDI.

Further, there was no significant difference between patients with relapsing–remitting MS and patients with progressive MS in any of the scores (|*t*| < 1.2, *p* > .35). On this basis, we focused our subsequent analyses on the whole group of patients with MS, independently of the disorder subtype.

### 3.2 Comparison of rsFC

Significantly lower interhemispheric **β**_**L**_‐ and **β**_**H**_‐band rsFC among nodes of the SMN was observed in patients with MS compared with healthy subjects (Figure 3; permutation *p* value = 5.45 × 10^−5^). Significant decrease in interhemispheric **β**_**H**_‐band rsFC was also found between secondary somatosensory cortex (S2) and contralateral frontal eye‐fields (FEFs), which were also interpreted as within‐SMN dysconnection rather than true cross‐network (i.e., SMN‐DAN) rsFC alterations. The effect was bilateral and most pronounced in the **β**_**H**_ band, where all significant connections were between each S2 and the contralateral central sulcus (CS), supplementary motor area (SMA), and frontal eye fields (FEF) as well as ipsilateral CS and SMA.

A similar analysis at the level of mean network rsFC confirmed the reduction of within‐SMN rsFC in the **β**_**H**_ band in patients compared with healthy subjects. It also revealed significantly lower **α**‐band rsFC within the DMN and between the DMN and two other RSNs, that is, the SMN and the LAN (Figure 4) in patients compared with healthy subjects (permutation *p* = 6.74 × 10^−5^). The fact that the nodewise rsFC contrast analysis (Figure 3) was not sensitive enough to detect reductions of DMN‐based rsFC suggests that the latter reflect genuinely network‐level effects. This hypothesis was further supported by the fact that **α**‐band DMN rsFC exhibited a larger effect size at the mean network level compared with nodewise connectivity. Indeed, the ratio of the effect size of mean network rsFC (i.e., group mean divided by SD), over the effect size of single connections averaged within or across each network, was well above one for intra‐DMN (7.8), cross DMN‐LAN (3.8), and cross DMN‐SMN (3.1) mean network rsFC in the **α** band but close to one (range: 0.9–1.25) for all other interactions and bands.

### 3.3 Correlation between rsFC and EDSS score in multiple sclerosis patients

The multiple regression between nodewise rsFC data in patients with MS and their EDSS score (Figure 5) highlighted a significant correlation for a subset of the band‐specific connections that were observed in the contrast analysis detailed in Section 3.2 (Figure 3), i.e., a significant correlation in the **β**_**H**_ band with nodes of the SMN (S2 and contralateral CS and FEF bilaterally) (permutation *p* = 3.12 × 10^−5^). All these significant correlations were negative, that is, lower rsFC was associated with higher disability status. Accordingly, the EDSS score was also significantly negatively correlated with mean network SMN rsFC in the **β**_**H**_ band as well in the **β**_**L**_ band.

### 3.4 Correlation between rsFC and disease duration in multiple sclerosis patients

Disease duration was negatively correlated with nodewise LAN rsFC within the **β**_**H**_ and across the **β**_**L**_ and **β**_**H**_ frequency bands (Figure 6; permutation *p* = 3.35 × 10^−4^). The significant correlations identified (i) connections within the frontal part of the LAN, that is, among three left frontal nodes (F3OPD: pars opercularis, LDIFG: left dorsal IFG, and F3TV: pars triangularis), and (ii) fronto‐temporal connections of the LAN between these three frontal nodes and two left temporal nodes (T1a/p: anterior/posterior superior temporal gyrus, LITG: left inferior temporal gyrus). Of note, the LITG was initially attributed to the DMN in de Pasquale et al. (2012), but considering its additional contribution to the verbal language network (Hickok & Poeppel, 2007), it was interpreted here as a within‐LAN connection rather than DMN‐LAN cross‐network interaction. Disease duration was also significantly negatively correlated with mean network LAN rsFC within and between **β**_**L**_ and **β**_**H**_ bands.

### 3.5 Correlations between rsFC and neuropsychological scores

Regression analyses of rsFC in patients with MS and healthy subjects disclosed significant correlations only with verbal fluency (COWAT score) and cognitive fatigue (cognitive part of the FSMC score) in both groups.

#### 3.5.1 Correlations between rsFC and verbal fluency

In patients, significant positive correlations of nodewise rsFC with the COWAT score identified connections between the LITG node and nodes of the LAN both within the **α** band (T1p and F3TV) and across the **α** and **β**_**L**_ bands (F3TV and T1p), and of the SMN (left CS, left S2) and DAN (left FEF) across the **α** and **β**_**L**_ bands (Figure 7, left column; permutation *p* = 1.33 × 10^−4^).

In healthy subjects, positive correlations with the COWAT score were also observed but involved different rsFC patterns, only within the **α** band, and all involving the posterior cingulate cortex (PCC) node of the DMN. COWAT scores correlated positively with connections between the PCC and nodes of the LAN (T1a, T1p, STS: superior temporal sulcus), the VAN (RMFG: right middle frontal gyrus, RSMG: right supramarginal gyrus), the DAN (LFEF) and the SMN (LCS) (Figure 7, right column; permutation *p* = 2.25 × 10^−4^).

Of note, these results appear to identify a topological reorganization of the brain‐behavior relation with verbal fluency associated with MS. This was confirmed by the statistical contrast between correlation matrices obtained in patients and healthy subjects, which demonstrated that correlations were significantly higher in patients compared with healthy subjects for the exact same connections as in Figure 7 (left), and significantly lower for the exact same connections than in Figure 7 (right) (permutation *p* = 8.74 × 10^−5^).

This relationship between rsFC and verbal fluency also allowed to characterize verbal fluency impairment as observed in MS patients (see Section 3.1) better than solely based on structural markers. Indeed, usage of the four mean network connectivity values that significantly discriminated between MS patients and healthy subjects (i.e., **α**‐band intra‐DMN, DMN‐LAN, and DMN‐SMN, and **β**_**H**_‐band intra‐SMN; see Section 3.2) in a regression model of verbal fluency significantly increased the explained variance compared to a purely structural regression (see Table 2).

**TABLE 2:** Key values from structuro‐functional model comparison

	*R* ^2^ (structural)	*R* ^2^ (structural, adjusted)	*R* ^2^ (structuro‐functional)	Model comparison *p* value
Verbal fluency	5.3%	9.5%	29.9%	3.5 10^−4^
Cognitive fatigue	3.4%	8.9%	24.5%	6.2 10^−4^

*Note:* Model parameter *R* ^2^ represents the percentage of explained variance of cognitive outcomes (Verbal fluency and Cognitive fatigue). *R* ^2^ (structural): Model with structural regressors only. *R* ^2^ (structural, adjusted): Structural model *R* ^2^ corrected for the number of additional dependent (functional) variables. Obtained as the mean of the null distribution generated by permutation of functional (but not structural) variables. *R* ^2^ (structuro‐functional): Full model including both structural and functional dependent variables. Model comparison *p* value: Null probability that *R* ^2^ values generated by permutation exceeds the observed structuro‐functional *R* ^2^.

#### 3.5.2 Correlations between rsFC and cognitive fatigue

In patients, negative correlations between nodewise rsFC and the cognitive part of the FSMC score were disclosed in the **δ**‐**β**_**H**_ cross‐frequency couplings between nodes of the LAN (F3TV, F3OPD) and nodes of the LAN (F3OPD, T1a, STS), the DMN (RMPFC: right medial prefrontal cortex, LITG, LAG: left angular gyrus) and the VAN (RVFC: right ventral frontal cortex, RMFG; Figure 8, left column; permutation *p* = 4.35 × 10^−4^).

In healthy subjects, negative correlations with the cognitive part of the FSMC score were also observed but involved different connections and frequency bands. Indeed, negative correlations were observed between **α**‐band posterior nodes of the DMN (PCC, LAG, RAG: right angular gyrus) and **α**‐ or **β**_**H**_‐band nodes of the DAN (L/RpIPS: left/right posterior intraparietal sulcus, L/RMT: left/right middle temporal gyrus), the VAN (RSTG: right superior temporal gyrus), the LAN (STS) and the SMN (L/RSMA), as well as the LITG (Figure 8, right column; permutation *p* = 3.7 × 10^−4^).

As for verbal fluency (Section 3.5.1), the apparent topological reorganization of brain‐behavior relation associated with MS was confirmed by considering correlation contrasts. This analysis demonstrated that correlations were significantly lower in patients compared with healthy subjects for the exact same connections than in Figure 8 (left), and significantly higher for the exact same connections than in Figure 8 (right) (permutation *p* = 1.03 × 10^−5^). Accordingly, regression modeling of cognitive fatigue based on neurostructural markers of MS was significantly improved by the inclusion of rsFC markers of MS (Table 2).

## 4 DISCUSSION

This MEG study involving a large population of patients with MS relied on a comprehensive analysis of functional connectivity at rest to characterize disease‐related changes in within‐ and cross‐frequency rsFC within and between major RSNs. Main original findings are that: (i) MS is characterized by reduced nodewise and mean network **β**‐band functional connectivity in the SMN, which is related to the level of disability, (ii) MS is associated with a reduced within‐ and cross‐network mean DMN rsFC in the **α** frequency band (cross‐network interactions involving the SMN and the LAN), (iii) disease duration is associated with reduced functional connectivity within the LAN in the **β** band, (iv) the DMN‐specific brain‐behavior correlates with verbal fluency and cognitive fatigue observed with multiple within‐ or cross‐frequency connections in healthy subjects is reorganized in patients with MS, and (v) the introduction of electrophysiological rsFC in a regression model of verbal fluency and cognitive fatigue in MS patients significantly increases the explained variance compared to a regression limited to structural MRI markers. Critically, all significant results were obtained after stringent corrections for multiple comparisons and after regressing out possible confounding effects of multiple variables (e.g., power of oscillatory brain activity, age, sex, educational level, MEG system type, and benzodiazepine status). These data therefore demonstrate that MS is associated with changes in RSNs that mainly involve within‐ and cross‐network interactions of the SMN, the LAN and the DMN. These changes represent robust neural correlates of specific behavioral and CIs observed in patients with MS.

### 4.1 Reduced sensorimotor network functional connectivity related to motor disability

In addition to a reduction in mean intra‐SMN **β**‐band functional connectivity, this study demonstrated lower functional connectivity in the lower and upper **β** frequency bands among nodes of the SMN in patients with MS compared to healthy subjects. The altered connections involved bilateral S2 with contralateral CS, SMA, and FEF. Crucially, a subset of the exact same node pair connections was (negatively) correlated with physical disability in patients as indexed by the EDSS. These findings therefore highly suggest that the observed difference in rsFC between patients and healthy subjects is driven by the sensorimotor disability classically observed in this disorder.

Importantly, none of the included patients had an EDSS score ≥ 6 (i.e., intermittent or unilateral constant assistance [cane, crutch or brace] required to walk 100 m with or without resting) and most were within the range of 2–5. This indicates that almost all included patients were able to walk unaided, and none were wheelchair‐bound. It therefore suggests that the observed reduction in SMN functional connectivity is not merely a consequence of sedentary living imposed by the disease. Still, we lack a specific measure of the patients' daily activity to confirm this hypothesis.

These results are in line with those of previous rs‐fMRI studies that have shown reduced rsFC between sensorimotor areas in MS (Eijlers et al., 2017; Filippi, Preziosa, & Rocca, 2019; Janssen et al., 2013; Richiardi et al., 2012; Rocca et al., 2012; Sbardella et al., 2015), which in some studies was correlated with disability severity (Janssen et al., 2013). Still, other studies have found either no difference (Dogonowski et al., 2014; Liu et al., 2011; Roosendaal et al., 2010) or even increased rsFC (Faivre et al., 2012; Roosendaal et al., 2010). Critically, studies showing increased rsFC involved patients with clinically isolated syndrome (Roosendaal et al., 2010) or weak physical disability (EDSS <1) (Faivre et al., 2012), which suggests that this finding is mainly observed at the early stages of the disease. A positive correlation between global **β**‐band MEG‐derived phase rsFC and MS‐related disability has also been reported (Tewarie et al., 2013), further suggesting that it represents an underlying electrophysiological correlate of the MS‐related sensorimotor disability.

The resting‐state electrophysiological SMN is known to be mainly driven by the **β**‐band power envelope correlation (Brookes et al., 2011; Hipp et al., 2012; Wens et al., 2014). Furthermore, synchronous **β**‐band oscillations in the sensorimotor cortices are important for active and ongoing control of coordinated movement and posture (Farmer, 1998; Rosanova et al., 2009). Considering the anatomical locations of the disconnected node pairs (i.e., S2, CS, SMA, and FEF), it might be hypothesized that such dysconnection could lead to impaired somatosensory‐motor integration (Forss & Jousmäki, 1998; Lin & Forss, 2002) in MS contributing to disability and altered motor performance (Arpin, Gehringer, Wilson, & Kurz, 2018; Cabib, Llufriu, Casanova‐Molla, Saiz, & Valls‐Solé, 2015). This hypothesis is in line with data showing that rehabilitation strategies specifically improving central integration of afferent proprioceptive inputs are more effective in improving balance disorders than conventional training in patients with MS with similar EDSS scores as those included in the present study (Gandolfi et al., 2015). These data suggest that electrophysiological rsFC could be a reliable method, free of performance bias, to properly assess the effects of therapeutic or rehabilitation strategies in MS.

### 4.2 Network‐level reduction of functional connectivity of the default‐mode network

Mean network functional connectivity corresponded to the average of the correlation strengths between node pairs belonging to a given RSN (i.e., mean within‐network rsFC) or between all nodes of one RSN and those of another RSN (i.e., mean cross‐network rsFC). It thus gave an estimate of the global rsFC level within or between the considered RSNs. This approach was used to reveal rsFC changes associated with MS that were subtle at the nodewise connectivity level but consistent across connections within or between RSNs. This was confirmed by the analysis comparing nodewise and mean network effect sizes, which showed a dramatic increase of the latter compared to the former especially for intra‐DMN, cross DMN‐SMN, and cross DMN‐LAN **α**‐band rsFC.

Significant decrease of **α**‐band mean network rsFC was found within the DMN, and between the DMN and the SMN/LAN in patients with MS compared to healthy subjects.

This MEG study therefore provides novel findings that complement previous fMRI studies (see *Introduction* for a summary) using an electrophysiological method that gives direct information about neural activity and is therefore free of any neurovascular coupling bias. Considering the clinical characteristics of our patients' cohort, it supports rs‐fMRI papers showing reduced DMN rsFC in patients with rather advanced multiple sclerosis. Indeed, it demonstrates that the disease‐related alterations in within‐ and cross‐network DMN rsFC actually involve a rather global disruption of within‐ and of some specific cross‐network (i.e., with the SMN and the LAN) DMN connections that is not detectable at the nodewise connectivity level. This concurs with the recognized role of the DMN as a core region for the functional integration with other RSNs (de Pasquale et al., 2012). It also demonstrates that these mean network rsFC alterations are specifically observed in the **α** frequency band, which is perfectly in line with the main carrying frequency of electrophysiological DMN rsFC previously reported (Brookes et al., 2011; Sjøgård et al., 2019; Vidaurre et al., 2018; Wens et al., 2014). These findings might also explain the more random (i.e., less structured and hierarchical) organization of functional brain networks that has been previously reported in **α**‐band phase‐based rsFC in patients with MS (Tewarie et al., 2013, 2014).

Finally, the reduction in mean within‐ and cross‐network DMN rsFC might be associated with the reorganization of the neural networks subtending verbal fluency and cognitive fatigue scores in patients with MS (see Section 4.4).

### 4.3 Disease duration is correlated with reduced language network functional connectivity

A strong association between disease duration and intrinsic functional connectivity within the LAN was observed in patients with MS in the **β** frequency band in the form of a negative correlation (i.e., the longer the disease duration, the lower the rsFC within the LAN). This was the case for both mean within‐LAN rsFC and for specific nodewise connections.

The occurrence and severity of language impairments in MS are actually poorly defined (for a review, see Renauld, Mohamed‐Saïd, & Macoir, 2016). Various verbal language impairments (picture naming, reading comprehension) as well as phonemic and semantic verbal fluency have been repeatedly reported in patients with MS (for a review, see Henry & Beatty, 2006). However, the heterogeneity of the methods used renders the elaboration of definite conclusions difficult (Renauld et al., 2016). Still, given the sensorimotor and various cognitive deficits characterizing MS, verbal language functions should be substantially affected (Renauld et al., 2016).

The strong negative correlation between disease duration and LAN rsFC found in this study might represent a neural correlate of the verbal language dysfunctions observed with the evolution of the disease. Unfortunately, no proper verbal language assessment was performed in our patients' cohort. Still, verbal fluency scores were significantly lower in patients than in healthy subjects and correlated with some language‐related network connections in patients. Although verbal fluency does not specifically assess verbal language function, some studies suggested that language processing is a critical component for this task (Whiteside et al., 2016). This finding therefore suggests that verbal language function might indeed be impaired in our patients' cohort. Still, disease duration did not significantly correlate with verbal fluency in our patient group (*r* = −.056, *p* = .163), which agrees with the fact that verbal fluency mainly assesses other cognitive (i.e., working memory, executive, and semantic memory) functions than verbal language. These correlation results stress the importance of behaviorally investigating verbal language function in MS. Such investigations will ultimately confirm whether the observed correlation is indeed actually driven by a progressive deterioration of verbal language along the course of the disease.

### 4.4 Reorganization of resting‐state networks correlated with verbal fluency and cognitive fatigue

Correlations between rsFC and cognitive/behavioral measures disclosed significant correlations only with verbal fluency (positive correlation) and cognitive fatigue (negative correlation) scores in both patients with MS and healthy subjects. Furthermore, these two scores were two of only three scores (i.e., decreased verbal fluency; increased cognitive fatigue; increased motor fatigue) showing significant difference between patients and healthy subjects. Strikingly, for both verbal fluency and cognitive fatigue, but not for motor fatigue, significant correlation patterns with rsFC were significantly different in healthy subjects compared with patients. In healthy subjects, correlation patterns mainly involved within‐ (cognitive fatigue) and cross‐network (cognitive fatigue and verbal fluency) DMN interactions. By contrast, in patients with MS, they mainly involved within‐ and cross‐network LAN interactions (if we consider the LITG a LAN node, as supported by Hickok & Poeppel, 2007).

As discussed in Section 4.3., verbal fluency is not a language‐specific cognitive measure but rather reflects the integrity of various high‐level cognitive functions such as working memory, executive functions and semantic cognition (Ralph, Jefferies, Patterson, & Rogers, 2017; Whiteside et al., 2016). In healthy subjects, we found a positive correlation between verbal fluency scores and the strength of PCC interactions with widespread nodes of the LAN, the SMN, the DAN, and the VAN. This result is in line with fMRI studies that disclosed a key role of the postero‐medial nodes of the DMN in verbal fluency (see, for example, Gauthier, Duyme, Zanca, & Capron, 2009; Shapira‐Lichter, Oren, Jacob, Gruberger, & Hendler, 2013; Dacosta‐Aguayo et al., 2015; Yin, Zhu, He, Li, & Li, 2015). By contrast, the correlation between verbal fluency and rsFC observed in patients with MS showed a significantly different correlation profile, which suggests the occurrence of disease‐related functional reorganization to sustain verbal fluency with recruitment of different network components than those observed in healthy subjects. These RSN configurations partly involved brain connections that can be attributed to lexico‐semantic processing/cognition (LITG‐F3TV, LITG‐T1P, for reviews, see for example, Hickok & Poeppel, 2007 or Ralph et al., 2017) at the detriment of the PCC involvement.

Cognitive fatigue is defined as the decrease in cognitive resources developing over time on sustained cognitive demands independently of sleepiness (Borragán, Slama, Destrebecqz, & Peigneux, 2016; Linnhoff et al., 2019). Cognitive fatigue will therefore impact the performance in several cognitive domains such as cognitive control, high‐level information processing, or sustained attention (Borragán et al., 2016). In healthy subjects, we found cognitive fatigue scores to be negatively correlated with the strength of within‐ and cross‐network posterior DMN interactions involving the DAN, VAN, LAN, and SMN, mostly centered around posterior regions of the DMN. As for verbal fluency, the correlation between cognitive fatigue and rsFC observed in patients showed a significantly different correlation profile that mainly involved connections between the left inferior frontal gyrus and nodes of the LAN, the DMN and the VAN. This finding demonstrates that the maintenance of cognitive resources in patients with MS rely on other network configurations than in healthy subjects with a reduced implication of within and cross‐network DMN interactions.

MS is often referred to as a structural disease (Compston & Coles, 2008; Mandolesi et al., 2015). For functional measures like MEG rsFC to be of additional utility as markers for MS‐related CI, they should add some information not already explained by the available structural measures. This study showed that, in fact, including functional connectivity in a regression model significantly added explanatory power over purely structural measures. Previous studies have shown a correlation between MEG rsFC and overall cognition (Tewarie et al., 2013), and we here show that it can independently explain a significant amount of variation in *specific* MS‐related CIs as well.

### 4.5 Methodological considerations and limitations

This study is the first to investigate rsFC in MS using band‐limited power envelope correlation, and as such, lacks direct comparability to the existing literature. All previous MEG/EEG studies of MS used phase‐based rsFC, which measures different aspects of electrophysiological brain interactions (Engel, Gerloff, Hilgetag, & Nolte, 2013). So, relating our results to the available electrophysiological literature is not straightforward. Still, there is a growing literature on the relationship between phase‐ and amplitude‐based coupling measures. They have been shown to be moderately to strongly correlated (Colclough et al., 2016; Siems & Siegel, 2020; Sjøgård et al., 2020) while still providing complementary, nonredundant information (Siems & Siegel, 2020). Furthermore, both of them have been shown to be related to fMRI rsFC in some ways (e.g., Tewarie et al., 2014, 2016). There is also evidence that, as rsFC based on power envelope correlation, phase coupling also displays some intrinsic (i.e., task independent) properties, although to a lesser degree than envelope correlation (Sjøgård et al., 2020). However, MEG power envelope correlation is closely related to rs‐fMRI functional connectivity, which is why we have split out discussion between the fMRI literature and the (phase‐based) MEG literature. That said, the fact that the neurovascular coupling seems to be altered in MS (Marshall et al., 2014) may also limit the validity of the comparison to fMRI. To mitigate this lack of comparability, we used here a large cohort of patients, a comprehensive analysis of a large‐scale multifrequency connectome, and a stringent control of confounding factors and false positives (as further discussed below).

It is noteworthy that, on general grounds, the interpretation of MS‐related rsFC increases or decreases is not straightforward. Both directions of change have been reported with fMRI rsFC and could either represent beneficial or maladaptive processes (Schoonheim, Meijer, & Geurts, 2015). Additionally, pathological white matter damage may lead to both increases and decreases in rsFC (Tewarie et al., 2018). That said, our MEG data consistently identified rsFC decreases only in patients with MS, and these decreases were related to worse clinical and cognitive scores.

Another limitation is that we only considered static rsFC estimates, which encapsulates the time‐averaged, temporally stable brain interactions over the course of minutes. This excludes dynamic features of rsFC (Baker et al., 2014; de Pasquale et al., 2012; Núñez et al., 2019; O'Neill et al., 2015; Seedat et al., 2020; Tewarie et al., 2019; Vidaurre et al., 2018; Wens et al., 2019). MS may also alter these dynamics, which might provide further insight into its physiopathology, as was shown in a comparison between static and dynamic rsFC in neurodevelopment (Hunt et al., 2018), although interpreting time‐dependent rsFC remains challenging (Hutchison et al., 2013). Results from our group in a similar participants' population demonstrated that transient brain dynamics is slightly altered in MS with a less dynamic frontal DMN in males with MS and a reduced activation of the same network in females with MS (Van Schependom et al., 2019). On the other hand, our current results are statistically robust (see discussion below).

It is also worth cautioning about a possible interpretational pitfall regarding the cross‐frequency approach (Brookes et al., 2016) due to our use of conventional frequency bands, which may not reflect the data at play in the rsFC changes reported here (although Vidaurre et al., 2018 showed that MEG data‐driven bands converge with the conventional ones). Cross‐frequency coupling identified across adjacent frequency bands could therefore merely reflect a broadband process overlapping the two bands and be artifactually coined as cross‐frequency. The **β**_**L**_‐**β**_**H**_ (Figure 6) and **α**‐**β**_**L**_ couplings (Figure 7) disclosed in our brain‐behavior correlations may be of that type. On the other hand, it is more likely that couplings across nonadjacent frequency bands such as the **δ**‐**β**_**H**_ and **α**‐**β**_**H**_ couplings disclosed in Figure 8 reflect genuinely cross‐frequency interactions. On a similar note, our rsFC analyses focused on the frequency bands typically considered to carry the electrophysiological RSNs, so we did not include gamma‐band rsFC (see, for example, Hipp et al., 2012). This means that some disease‐related rsFC changes might have been missed, either within the gamma band or between the lower frequency bands and the gamma band.

Last, and critically, the results obtained in this study generally err on the conservative side and may thus be fraught with false negatives. First, the low‐density, 32‐node brain parcellation used here was limited to nodes of well‐known RSNs (based on a meta‐analysis of fMRI rsFC, as in de Pasquale et al., 2012). Although the spatial smoothness inherent to MEG partially mitigates this coarseness, some brain areas were poorly sampled at best in our connectome analysis (see Figure 2a) and functional connections outside these RSN regions were not considered here. Further, a connectome design based on fMRI may not be optimal to investigate the multi‐spectral signatures of rsFC, to which fMRI is insensitive. Given the close anatomical correspondence between electrophysiological and fMRI RSNs (see, for example, Brookes et al., 2011), using this type of design is well justified in the context of within‐frequency rsFC but it may have a more limited value for cross‐frequency couplings. These two limitations mean that our analyses might miss MS‐related alterations in network configurations outside these RSNs. Second, our statistical design included stringent control for several confounding factors, and also for the large number of comparisons (496 connections for nodewise rsFC or 15 for mean network rsFC estimates, 10 pairs of frequency bands, 38 tests) so as to ensure a family‐wise false positive rate below 5%. The price to pay is a lessened sensitivity to true differences and correlations. This was presumably mitigated by our inclusion of a large number of participants, and the negative impact of our unbalanced design in contrasts was further alleviated by the use of Welch's *t* statistic. Still, given the lack of comparable works, we chose to focus on conservative statistics, without overinterpreting nonsignificance as a lack of genuine effect. The results reported here thus represent the most robust effects of MS on electrophysiological RSNs and its brain‐behavior correlates.

### 4.6 Conclusion

This MEG study demonstrates that MS entails several robust frequency‐specific network‐level and regional changes within and between RSNs (mainly the SMN, DMN, and LAN) that are related to motor disability, disease duration and specific CIs. It also shows that MEG rsFC relying on power envelope correlation brings significant independent information in *specific* MS‐related CIs outside structural brain abnormalities. This shows that frequency‐specific RSN changes may be suitable candidates for electrophysiological markers of both clinical and cognitive aspects of the disease with the remarkable advantages of being totally noninvasive, free of performance bias and free of any neurovascular issue. The ability of EEG to uncover similar RSNs as MEG (Coquelet et al., 2020; Liu et al., 2017; Siems et al., 2016; Sockeel et al., 2016) should facilitate the dissemination of the proposed approach in MS and other brain disorders.

## Data Availability

Data and code used for this research project will be shared in the context of an academic and scientific collaboration formalized by a collaboration agreement accepted by the Université Libre de Bruxelles and the Vrije Universiteit Brussel. Please contact the corresponding author for any inquiries.
